# Protective Effect of the Aqueous Extract of *Deschampsia antarctica* (EDAFENCE^®^) on Skin Cells against Blue Light Emitted from Digital Devices

**DOI:** 10.3390/ijms21030988

**Published:** 2020-02-02

**Authors:** Silvia Lorrio, Azahara Rodríguez-Luna, Pablo Delgado-Wicke, Marta Mascaraque, María Gallego, Azahara Pérez-Davó, Salvador González, Ángeles Juarranz

**Affiliations:** 1 Department of Biology, Faculty of Sciences, Autónoma University of Madrid (UAM), Instituto Ramón y Cajal de Investigación Sanitaria (IRYCIS), 28049 Madrid, Spain; silvia.lorrio@gmail.com (S.L.); pablo.delgado@uam.es (P.D.-W.); marta.mascaraque@uam.es (M.M.); mgallegoah@gmail.com (M.G.); angeles.juarranz@uam.es (Á.J.); 2 Medical Affairs Department, Cantabria Labs, 28043 Madrid, Spain; azahara.rodriguez@cantabrialabs.es (A.R.-L); azahara.perez@cantabrialabs.es (A.P.-D.); 3 Department of Medicine and Medical Specialties, Alcalá de Henares University, 28805 Madrid, Spain

**Keywords:** artificial blue light, hyperpigmentation, reactive oxygen species, natural extract, dermal fibroblast, melanocyte

## Abstract

Skin is being increasingly exposed to artificial blue light due to the extensive use of electronic devices. This, together with recent observations reporting that blue light—also known as high-energy visible light—can exert cytotoxic effects associated with oxidative stress and promote hyperpigmentation, has sparked interest in blue light and its potential harmful effects on skin. The photoprotective properties of new extracts of different botanicals with antioxidant activity are therefore being studied. *Deschampsia antarctica* (Edafence^®^, EDA), a natural aqueous extract, has shown keratinocyte and fibroblast cell protection effects against ultraviolet radiation and dioxin toxicity. In this regard, we studied the protective capacity of EDA against the deleterious effects of artificial blue light irradiation in human dermal fibroblasts (HDF) and melanocytes. We analyzed the impact of EDA on viability, cell morphology, oxidative stress, melanogenic signaling pathway activation and hyperpigmentation in HDF and melanocytes subjected to artificial blue light irradiation. Our results show that EDA protects against cell damage caused by artificial blue light, decreasing oxidative stress, melanogenic signaling pathway activation and hyperpigmentation caused by blue light irradiation. All these findings suggest that EDA might help prevent skin damage produced by artificial blue light exposure from screen of electronic devices.

## 1. Introduction

The skin is the main defensive barrier in the body against a large variety of environmental factors and is responsible for maintaining body homeostasis, defence and repair [1]. It is well known that exposure to external factors (e.g., sun radiation, air pollution, tobacco smoke, nutrition and temperature) and internal factors (e.g., sleep deprivation or stress) trigger molecular processes that damage skin structure [2,3].

The terrestrial sunlight spectrum consists of short, high-energy wavelengths, from ultraviolet radiation (UV, 280–400 nm) to visible light (VIS, 400–700 nm), and long, low energy wavelengths: infrared radiation (700 nm–1 mm). UV radiation, which is responsible for the majority of harmful effects on the skin, represents just 2%–5% of the terrestrial sunlight [2]. The VIS spectrum represents approximately 50% of the solar spectrum arriving on the earth’s surface, but it was considered to have little impact on skin due to its low energy [4] and hence received little attention. However, recent observations of skin photodamage following VIS exposure and prevention strategies have sparked increased interest [5,6].

VIS is a solar spectrum radiation range that can be classified according to its emission wavelength into blue (400–495 nm), green (495–590 nm) and red light (590–700 nm) (Figure S1A). VIS wavelengths penetrate into the deepest parts of the dermis reaching the different types of skin cells. Although the medical use of VIS has been in practice for a long time, a recent investigation confirmed that dermal fibroblasts are damaged by exposure to this radiation range, being the skin cells that show greater changes as opposed to keratinocytes or melanocytes [7]. Artificial VIS induces cell oxidative stress, damage and dysfunction, causing signs of early photo-aging [4,8,9]. In addition, VIS-induced oxidative stress can also alter metalloproteinase activities and decrease collagen production [10]. Several studies report that blue, green and red light can have very different effects on skin cells. Red light irradiation enhances cell growth, synthesis of procollagen I, and skin homeostasis [11,12]. In contrast, blue light, also known as high-energy visible light, exerts anti-proliferative and oxidative stress-associated effects [13,14], disrupts epidermal permeability barrier [15], and promotes pronounced and longer-lasting hyperpigmentation [5]. Green and red wavelengths are thus considered less cytotoxic and damaging than blue light. In addition to the sun, there are multiple artificial sources of blue light, including electronic devices, fluorescent lamps or light-emitted diodes (LED). Benefits from controlled LED therapy are observed in the treatment of a number of dermatological diseases, due to its potential photodynamic and photobiological effect [16]. Nevertheless, many scientific studies also report on the potential harmful effect of VIS, where blue light is potentially more harmful than red and green wavelengths.

In agreement with these findings, extensive exposure times to artificial sources of blue light through the use of electronic devices can be considered to cause cellular alterations and oxidative stress, harming skin cells [4]. The key point in determining the real skin damage is the accumulative dose of natural and artificial light during the day, plus the increasing nighttime exposure to artificial light emitted by electronic devices’ screens. Therefore, further studies taking into consideration more parameters apart from the wavelengths, such as total received doses, irradiance and chronic exposure times, are necessary to evaluate the real accumulative damage to skin [17].

In this regard, a standardized aqueous extract of *Deschampsia antarctica*—EDA—might protect the skin against some of the aforementioned exposure factors which induce skin aging. *Deschampsia antarctica*—a polyextremophile Gramineae—is a flowering plant native to Antarctica which is capable of thriving under extremely low temperatures, high concentrations of oxygen, high solar radiation, extreme dryness and high salinity. Due to the extreme conditions, this Gramineae has developed several secondary metabolic routes which produce phenolic substances with specific keratinocyte and fibroblast cell protection effects, as well as DNA damage prevention effects and downstream responses against both UV and dioxin toxicity, as previously reported by our group [7,8].

In this work, we studied the protective effect of EDA against significant molecular and cellular changes in normal fibroblasts and melanocytes exposed to blue light from artificial electronic devices. In particular, we observed mitochondrial damage, production of reactive oxygen species (ROS), and pigmentation induction, including mitogen-activated protein kinases (MAPKs) pathway activation. These parameters would accurately demonstrate EDA’s ability to protect normal cellular function and therefore minimize premature skin aging.

## 2. Results

### 2.1. EDA Promotes Cell Viability Reduced by Artificial Blue Light Exposure

**Figure 1:**
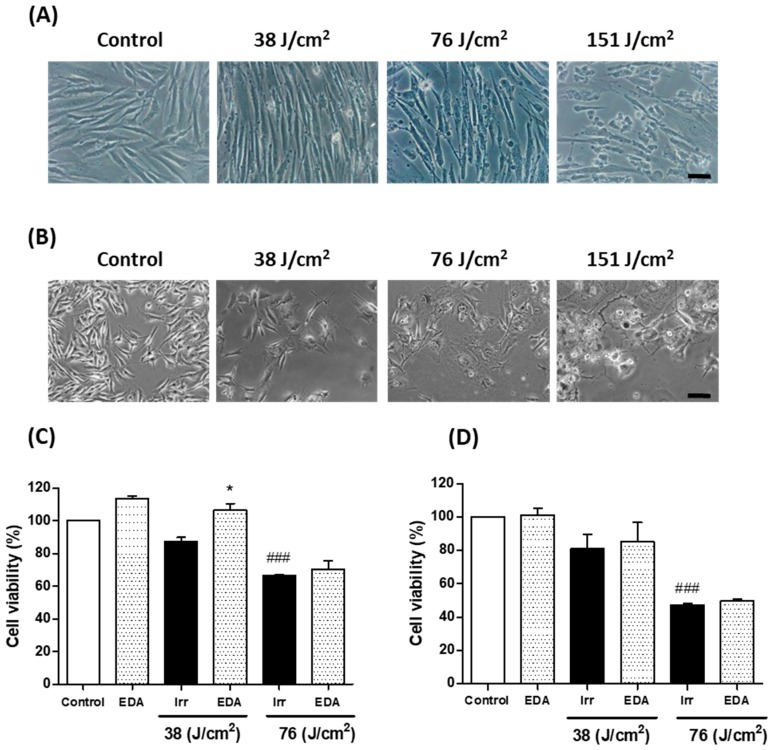
Cell viability changes induced in HDF (**A**, **C**) and melanocytes (**B**, **D**) exposed to artificial blue light and EDA pre-treatment. Cells were incubated with EDA 0.1 mg/mL for 24 h and then irradiated at the indicated doses. Cell viability was evaluated by MTT assay performed 24 h after irradiation (*n* ≥ 3). Data were expressed as % of control cells. Data are shown as mean ± standard error of the mean (SEM). ^###^ *p* < 0.001 vs control cells and * *p* < 0.05 vs the corresponding irradiated (Irr) cells. Scale bar: 50 µm.

In order to evaluate cell viability after blue light exposure, we tested three different irradiation doses (38, 76 and 151 J/cm^2^) in HDF. Cell viability decreased with increasing blue light doses. Based on these results, we selected 38 J/cm^2^ as the appropriate dose for mitochondrial damage and pigmentation experiments as it induced relatively low lethal oxidative stress-associated effects (Figure 1). We evaluated the effect of EDA on the cell viability of HDF and murine melanocytes. EDA 0.1 mg/mL significantly increased cell viability in HDF irradiated at 38 J/cm^2^ measured by 3-[4,5-dimethylthiazol-2-yl]-2,5-diphenyltetrazoliumbromide (MTT). A modest increase was also observed in EDA-treated cells exposed to 76 J/cm^2^ 24 h after irradiation. These results were corroborated in melanocytes, where cell viability also decreased after blue light irradiation, and a modest increase was observed in cells treated with EDA.

### 2.2. EDA Prevents Oxidative Stress Induced by Artificial Blue Light Irradiation

**Figure 2:**
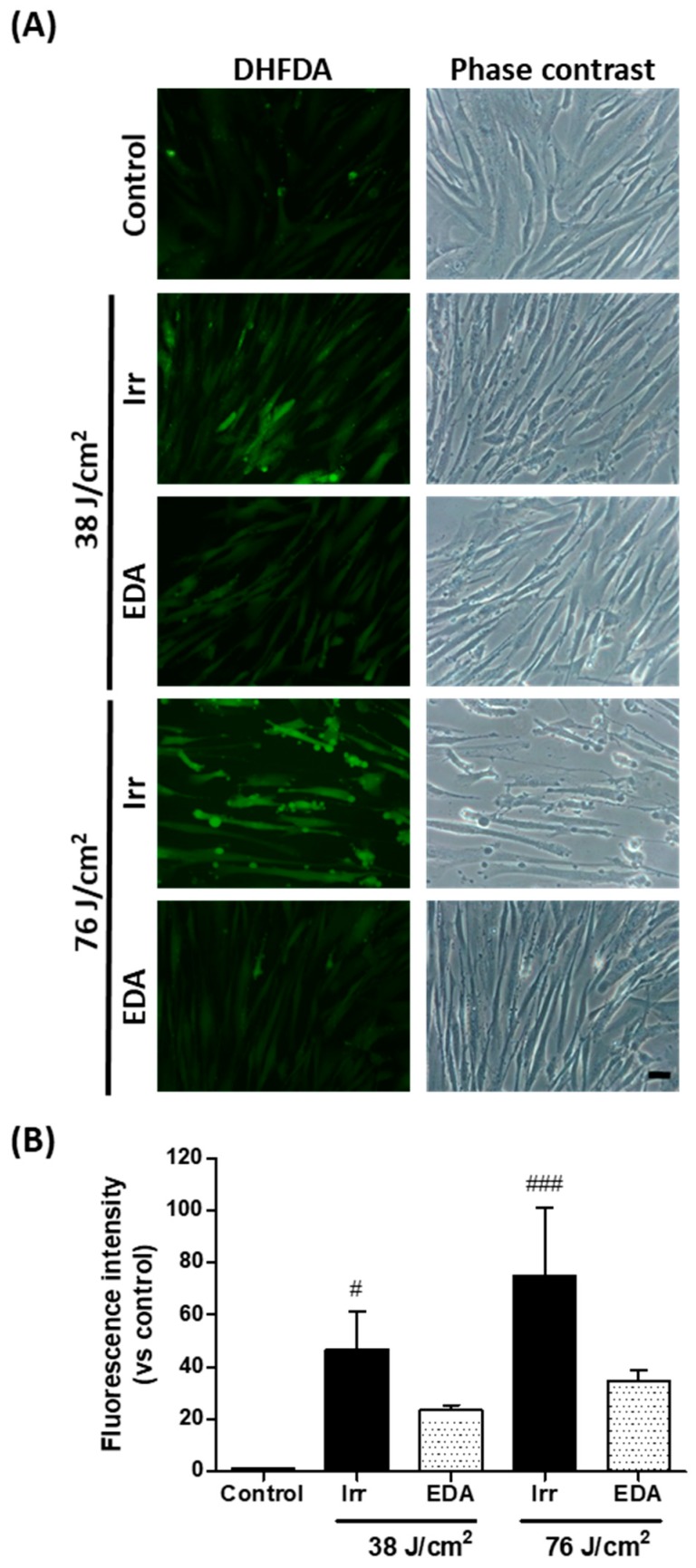
Oxidative stress in human fibroblasts exposed to artificial blue light and EDA pre-treatment. Oxidative stress was evaluated by DHFDA assay. Cells were incubated with EDA 0.1 mg/mL for 24 h, loaded with DHFDA, irradiated at 38 and 76 J/cm^2^, washed and observed under the microscope immediately after irradiation. ROS are evidenced by green fluorescence in HDF exposed to artificial blue light and EDA (**A**). Quantification of DHFDA by fluorescence (*n* ≥ 5) (**B**). Data are shown as mean ±SEM. ^#^ *p* < 0.05, ^###^ *p* < 0.001 vs control. Scale bar: 50 µm.

We evaluated oxidative stress in HDF exposed to blue light, using dihydrofluorescein diacetate (DHFDA), by fluorescence microscopy. Figure 2 shows a stronger DHFDA signal in cells exposed to blue light as compared to control cells, corroborating that artificial blue light irradiation increased intracellular oxidative stress in a dose-dependent manner. In addition, phase contrast microscopy showed altered cell morphology proportional to the light dose employed, and cell membrane deformations as blebs were observed particularly after the dose of 76 J/cm^2^ (Figure 2A). EDA reduced ROS production as the DHFDA signal was diminished under the fluorescence microscope. In addition, cell morphology was protected by EDA, as observed under the phase contrast microscope. Measurement of DHFDA fluorescence intensity confirmed the above findings (Figure 2B).

### 2.3. EDA Prevents Blue Light-Induced Alterations on Mitochondrial Morphology and Membrane Potential

**Figure 3:**
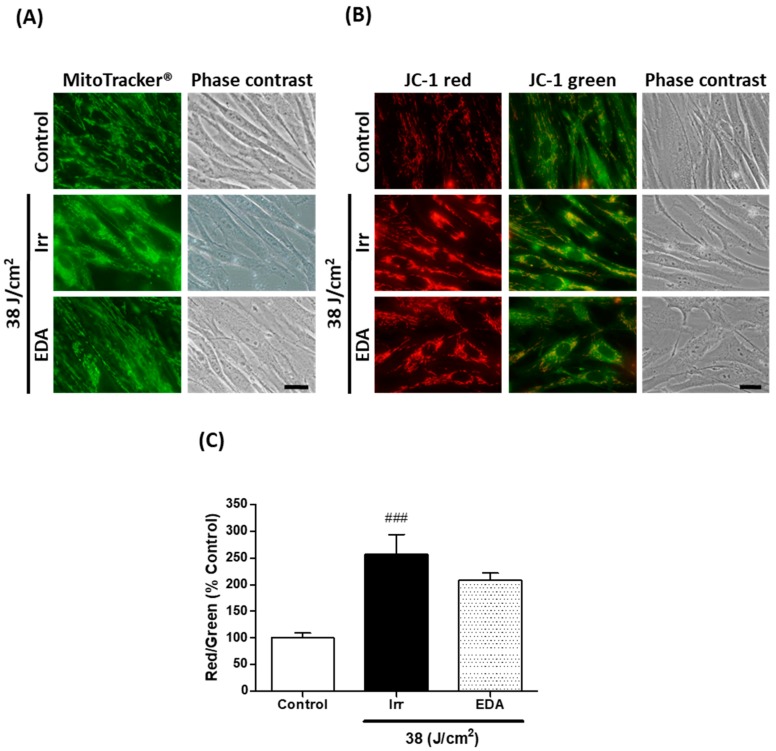
Mitochondrial morphology and membrane potential in human fibroblasts exposed to artificial blue light and EDA pre-treatment. Cells were incubated with EDA 0.1 mg/mL for 24 h, irradiated at 38 J/cm^2^ and incubated with MitoTracker^®^ or loaded with JC-1 to be observed under the microscope 24 h after irradiation. Mitochondrial morphology was documented using the fluorescent probe MitoTracker^®^ (**A**). Mitochondrial membrane potential was evaluated with the dye JC-1 (**B**). Quantification of JC-1 red/green fluorescence ratio (*n* ≥ 5) (**C**). Data are shown as mean ±SEM. ^###^ *p* < 0.001 vs control. Scale bar: 20 µm.

We used the fluorescent probe MitoTracker^®^ green to document mitochondrial morphology (Figure 3A). Non-irradiated control HDF presented elongated mitochondria homogeneously distributed throughout the cells’ cytoplasm. Mitochondria of HDF irradiated at 38 J/cm^2^, on the other hand, were much shorter or even showed a round-shaped, almost granulated appearance. Cells treated with EDA prior to irradiation were mostly elongated, showing protection against the damaging effects of blue light on mitochondria morphology.

The effect of blue light irradiation on mitochondrial membrane potential was evaluated using the indicator dye 5,5,6,6’-tetrachloro-1,1’,3,3’-tetraethylbenzimi-dazoylcarbocyanine iodide (JC-1) (Figure 3B). The red/green fluorescence ratio of JC-1 can be considered a direct indication of the state of mitochondria membrane potential [18]. We observed that blue light exposure induced a significant increase in the red/green fluorescence ratio compared to control, indicating mitochondrial hyperpolarization (Figure 3C). Cells pre-treated with EDA showed a modest decrease in the red/green ratio.

### 2.4. EDA Regulates Artificial Blue Light-Induced Pigmentation through p38 Melanogenic Signalling Pathway Modulation

**Figure 4:**
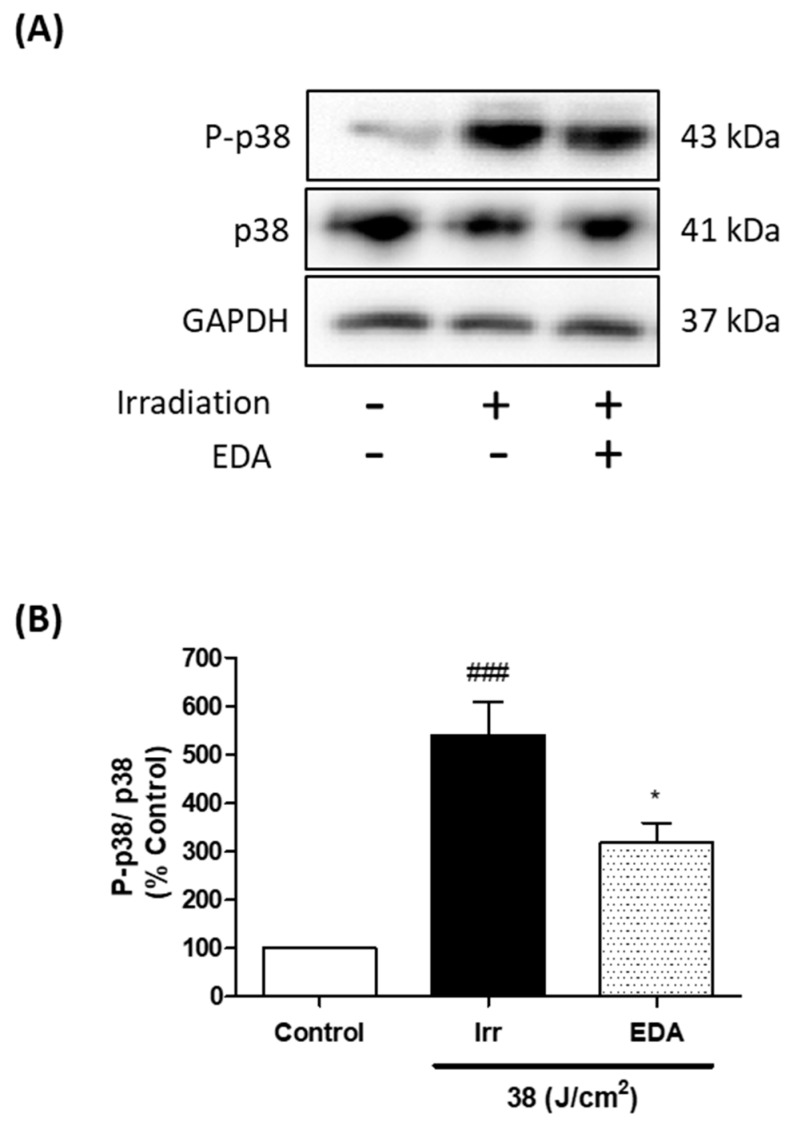
Phosphorylation of p38 in HDF exposed to artificial blue light and EDA pre-treatment. Phosphorylation of p38 was evaluated by WB. Cells were incubated with EDA 0.1 mg/mL for 24 h, irradiated and proteins extracted 1 h after irradiation. Representative immunoblots (**A**) and quantification plot (**B**) are shown (*n* ≥ 3). Data were expressed as % of control cells. Data are shown as mean ± SEM. ^###^ *p* < 0.001 vs control; * *p* < 0.05 vs irradiated cells.

VIS affects skin cells, especially dermal fibroblasts [7] which are involved in both physiological and pathological skin pigmentation, acting on melanocytes by secreting a large number of proteins, cytokines and growth factors [19]. We studied p38 melanogenic signalling pathway activation through a Western blot analysis. Artificial blue light induced phosphorylation, and therefore activation of p38. Treatment with EDA significantly reduced p38 phosphorylation 1 h after blue light irradiation (Figure 4).

### 2.5. EDA Prevents Hyperpigmentation Induced by Artificial Blue Light

**Figure 5:**
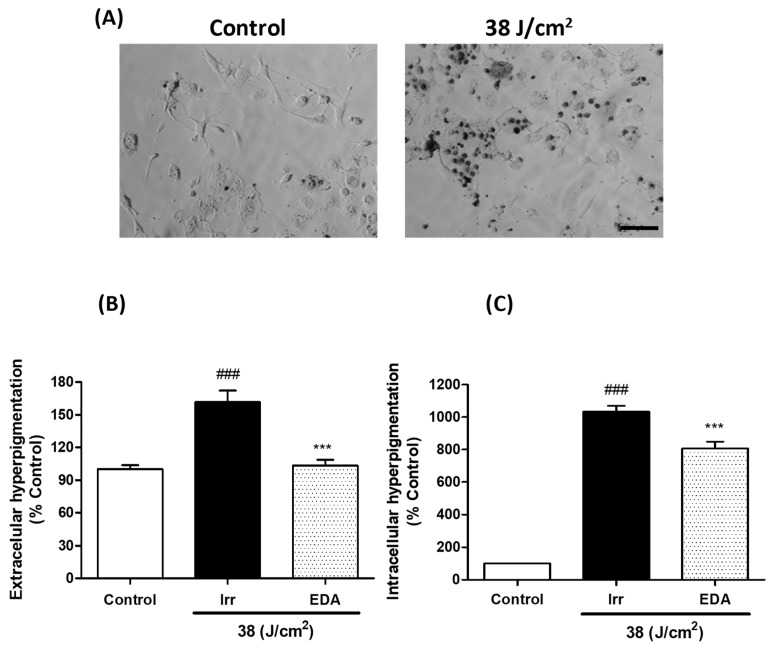
Extracellular and intracellular hyperpigmentation in melanocytes exposed to artificial blue light and pre-treatment with EDA. Microphotographs show how control and irradiated melanocytes have different amounts of melanin dark granules (**A**). Cells were incubated with EDA 0.1 mg/mL for 24 h, irradiated (38 J/cm^2^), fresh medium replaced and extracellular (**B**) and intracellular (**C**) melanin pigments collected, solubilized and quantified 3 h later by absorbance measurements (*n* ≥ 4). Data were normalized by mg protein and expressed as % of control cells. Data are shown as mean ±SEM. ^###^ *p* < 0.001 vs control; *** *p* < 0.001 vs irradiated. Scale bar: 50 µm.

We evaluated hyperpigmentation in melanocytes exposed to artificial blue light. Blue light irradiation induced a significant darkening of both extracellular and intracellular melanin pigments 3 h after irradiation. Treatment with EDA significantly reduced hyperpigmentation (Figure 5).

## 3. Discussion

The amount of time spent in front of digital devices is continually on the rise, whether it be computers, mobile phones, tablets or television; giving birth to the term “screentime”. The negative effects of screentime are related to disturbances such as irritability, cognitive disorders and obesity, to mention just a few [20]. This phenomenon of our modern age—also called digital pollution—is shaking the concept of healthy aging, and several studies have focussed on this area, identifying urban-associated diseases [21,22,23]. In the same way, the interest in studying the role of artificial VIS in skin aging is increasing, and several studies report oxidative stress, damage and mitochondria dysfunction induced by artificial VIS in skin cells [4,8,9]. The emission spectrum of several digital devices was reviewed by Rascalou et al., and they determined their blue light irradiance to be 36 μW/cm^2^ [1]. Passeron’s group also reported that the irradiance of blue light from the most potent TV screens is 30 µW/cm^2^ [17]. Since we wanted to simulate chronic exposure to digital devices but cells cannot be irradiated for long periods, we used a blue light source with higher irradiance (42.05 mW/cm^2^). Cells were irradiated with doses equivalent to spending 290 h—or 6 h a day for 7 weeks—in front of digital devices.

Artificial blue light induced a decrease in cell viability, which was more pronounced in melanocytes than in HDFs. Melanin plays an important role in protecting the skin against UV radiation. However, melanin is also involved in post-exposure skin damage. Premi et al. [24] showed that UV-induced reactive oxygen and nitrogen species excite an electron in fragments of the pigment melanin in melanocytes. Excited melanin then transfers the energy to DNA inducing cyclobutane pyrimidine dimers (CPD) formation, even more than 3 h after UV exposure. These “dark CPD” induce DNA mutations and cell apoptosis. Melanin-related DNA damage via CPD formation could therefore be playing a role in blue light-induced cell death in melanocytes.

Currently, it is well demonstrated that VIS induces hyperpigmentation, with shorter wavelengths being the main inductors of the pigmentation pathway [5]. Thereby, Passeron’s group studied visible light-induced hyperpigmentation by activation of opsin 3 pathway, which induces a calcium influx and activates ERK and p38, among others, producing the phosphorylation of microphthalmia-associated transcription factor (MITF), raising tyrosinase activity, and finally increasing melanin production in cells [25]. In this study, we showed that blue light simulating a chronic exposure to digital devices, induced oxidative stress in HDF, damaged mitochondria by changing their morphology and membrane potential, and increased hyperpigmentation. Maeda et al. [26] reported that the photoxidation of melanogenic precursors and further metabolites after UVA radiation might be the mechanism responsible for skin persistent pigmentation without reddening after exposure to sunlight. Likewise, photooxidation rather than melanin production might be the mechanism involved in hyperpigmentation after blue light irradiation in our experiments, since we observed hyperpigmentation as early as 3 h after irradiation. Further investigation to explore this point may be warranted.

Botanical extracts are particularly interesting to prevent the aforementioned skin alterations, as they are usually characterised by low toxicity and anti-stressor activity, and frequently provide several synergies such as anti-inflammatory, antioxidant and tissue repair activity. In this sense, EDA has previously been shown to provide significant protective activity against both UV and dioxin-induced damage, as well as against cellular senescence, amongst others [27]. Additionally, EDA in combination with antioxidants and retinoids demonstrated antiaging activity by improving skin barrier function, evening out skin tone and counteracting oxidative stress in the skin of women living in polluted urban zones [28,29].

Here, we demonstrated the protective activity of EDA in blue light-irradiated skin cells simulating chronic exposure to digital devices by preventing oxidative stress. Exposure to blue light also increased cell damage and morphology alteration, which were prevented by EDA pre-treatment. Previously, Ortiz-Espín et al. [30] have shown EDA’s ability to protect young human fibroblasts exposed to H_2_O_2_, increasing cell survival and anti-senescence-related markers expression such as sirtuin 1 and lamin A/C, and reducing the expression of the senescence-associated β-galactosidase protein after 24 and 48 h pre-treatment. In mitochondria, an increase in free radicals is induced during senescence, leading to oxidative stress in most cell compartments [31]. This imbalance is offset by redox proteins such as thioredoxins (Trxs) which decrease free radical-induced damage in stressed cells [32]. However, our prior study confirmed that Trx2 modulation was not involved in EDA’s protective effect [30]. Therefore, in this study we examined the effects of EDA involvement in structural functions linked to cytoplasm and mitochondrial morphology protection and the prevention of mitochondrial membrane potential alterations induced by blue light exposure. Mitochondrial disruption usually includes mitochondrial membrane depolarization, cytochrome c release into the cytosol and—finally—cell death. In this study, JC-1 red/green ratio in irradiated cells suggests that mitochondrial disruption includes hyperpolarization rather than depolarization, which is in consonance with using non-lethal irradiation doses. Nevertheless, EDA pre-treatment prevented mitochondrial membrane disruption, and a healthy mitochondrial morphology was corroborated with MitoTracker^®^. Since oxidative stress and mitochondrial dysfunction are known to play an important role in photo-aging, EDA might be a good candidate to prevent oxidative and mitochondrial damage—thus promoting healthy cell aging.

In terms of pigmentation, fibroblasts have a dynamic role in signal cross-talk between melanocytes and keratinocytes. Different studies have been published regarding the MAPK pathway involvement in melanogenesis, showing that hyperpigmentation can be induced by phosphorylation of MAPK p38, leading to the up-regulation of MITF and consequently increasing tyrosinase expression and hyperpigmentation [33,34]. In our study, blue light increased p38 phosphorylation in fibroblasts and hyperpigmentation was clearly induced in melanocytes exposed to artificial blue light. We observed in this study that EDA pre-treatment reduced the phosphorylation of p38 and decreased hyperpigmentation in fibroblasts and melanocytes exposed to blue light. In summary, these results support that treatment with EDA is a potential therapeutic agent that could slow skin aging by protecting skin cells from artificial blue light from digital devices, counteracting premature signs of photo-aging.

## 4. Materials and Methods

### 4.1. Cell Culture

HDF were obtained from a skin biopsy after trypsinization with 0.25% Trypsin-EDTA. B16-F10 mouse melanocyte cell line was kindly provided by Dr. Benilde Jiménez Cuenca, Instituto de Investigaciones Biomédicas «Alberto Sols» UAM-CSIC. Both HDF and melanocytes were cultured in Dulbecco’s modified eagle medium (DMEM) supplemented with 1% (*v*/*v*) penicillin G (100 U/mL) and streptomycin (100 µg/mL) and 10% (*v*/*v*) fetal bovine serum (FBS) (HyClone Laboratories, South Logan, UT, USA). Cells were maintained at 37 °C, 5% humidity and 5% CO_2_ in an incubator (Heraeus HERAcell, Thermo Scientific, Waltham, MA, USA).

### 4.2. Natural Extract and Cell Treatment

EDA is a hydrophilic extract from the leaves of *Deschampsia antarctica*, and was obtained as lyophilized powder from Cantabria Labs, Madrid, Spain. Stock solution was prepared in distilled water at a concentration of 10 mg/mL. The stock was then diluted in phenol red-free DMEM 1% FBS to the desired final concentration. Previous studies testing the efficacy of the extract against UV and dioxin reported concentrations ranging from 0.1 to 0.3 mg/mL [27]. Based on those studies, we determined to incubate cells with 0.1 mg/mL of EDA for 24 h before irradiation.

### 4.3. Irradiation

Fibroblasts and melanocytes were subjected to artificial blue light irradiation with the aim of studying the effects of the high energy visible light emitted by the screens of electronic devices [4]. The blue-light source was a narrow-band LED lamp, which emits light of 450–465 nm wavelength and 42.05 mW/cm^2^ power (Segainvex, Madrid, Spain) (Figure S1B). Blue light irradiation doses were 38, 76 and 151 J/cm^2^, which are equivalent to 15, 30 and 60 min of in vitro blue light exposition. Cells were irradiated in phenol red-free DMEM 1% FBS with the treatments. The light source was placed under the culture plates so that cells were irradiated directly from below, avoiding any possible shielding effects exerted by the culture medium or the treatments. Immediately after irradiation, fresh medium was added, and the cells were maintained in the incubator.

### 4.4. MTT Cell Viability Assay

Cell viability 24 h after irradiation or after removal of the treatments was determined using the MTT assay. MTT solution (100 μg/mL) was added to cell cultures and incubated for 3 h at 37°C. The resulting formazan precipitate was dissolved in dimethylsulfoxide (Panreac, Barcelona, Spain) and absorbance was measured at 542 nm using a plate reader (SpectraFluor, Tecan, Zürich, Switzerland). Data were normalized with respect to non-irradiated control values.

### 4.5. Reactive Oxygen Species Measurement

The cell’s production of ROS was determined by fluorescence microscopy using the fluorescent probe DHFDA [35]. Cells were incubated with the treatments for 24 h at the indicated concentrations and then loaded with DHFDA (Abcam, Cambridge, UK) to a final concentration of 7.5 μM for 50 min at 37 °C. Cells were subjected to blue light irradiation in the presence of the dye. DHFDA crosses the cell membrane and is hydrolysed by intracellular esterases to the non-fluorescent form, dihydrofluorescein; the latter reacts with intracellular ROS to form the fluorescent dye, fluorescein. Immediately after irradiation, cells were directly analysed by fluorescence microscopy.

### 4.6. Mitochondrial Morphology and Membrane Potential

To assess mitochondrial morphology, we used the fluorescent probe MitoTracker^®^ green (Invitrogen, Thermo Fisher Scientific, Waltham, MA, USA). After irradiation, the cells were incubated with 1 μg/mL for 15 min at 37°C. To assess the mitochondrial membrane potential we used JC-1 dye (Invitrogen). After irradiation, cells were incubated with 1 μg/mL for 30 min at 37 °C. JC-1 is a green fluorescent dye that can cross cell membranes, accumulating inside negatively-charged mitochondria in a potential-dependent manner, shifting its fluorescence emission to red due to red fluorescent J-aggregates formation. Consequently, the red/green fluorescence intensity ratio is an indication of membrane potential. Mitochondrial depolarization produces a decrease in the red/green fluorescence intensity ratio, as compared to control mitochondria. Microscopic images were obtained using the epifluorescence microscope with blue or green excitation filters.

### 4.7. Protein Electrophoresis and Western Blot

Cell cultures were washed with cold PBS and then incubated in RIPA buffer with Triton, pH 7.4 (bioWORLD, Dublin, OH, USA), containing protease (complete ULTRA tablets Mini EDTA-free EASYpack, Roche, Mannheim, Germany) and phosphatase (PhosSTOP EASYpack, Roche) inhibitors. Protein concentration was determined using the Pierce™ BCA Protein Assay Kit (Thermo Fisher Scientific). Protein extracts were diluted in Laemmli buffer (Bio-Rad, Hercules, CA, USA) and heated for 5 min at 98 °C. A Mini-PROTEAN cell was used for electrophoresis in acrylamide/bisacrylamide gels in denaturing conditions (SDS-PAGE). Protein transfer onto PVDF membranes (Bio-Rad) was then performed with a Transblot Turbo system (Bio-Rad). Membranes were blocked with 5% skimmed milk in 0.1% TBS-Tween 20, and then incubated with primary antibodies anti-p38, anti-phospho-p38 (both from Cell Signaling Technology, Danvers, MA, USA), and anti-GAPDH (Abcam), and peroxidase-conjugated secondary antibodies (GE Healthcare, Chicago, IL, USA). Protein bands were visualized by chemiluminiscence (Pierce ECL Plus Kit, Thermo Fisher Scientific) using an image acquisition system (ChemiDoc XRS+, Bio-Rad), and analyzed using Image Lab version 2.0.1 software (Bio-Rad). Data were normalized with respect to the loading control and then to the non-irradiated control values.

### 4.8. Melanin Content Determination

Hyperpigmentation was measured following the protocol described by Bellei et al., 2008 [36], with some modifications. Cell culture medium was collected for extracellular melanin pigments measurement. Cells were washed with cold PBS and lysed with RIPA buffer with Triton, pH 7.4 (Bioworld), containing protease (complete ULTRA tablets Mini EDTA-free EASYpack, Roche) and phosphatase (PhosSTOP EASYpack, Roche) inhibitors. Lysates were then centrifuged at 17,000 g for 10 min at 4°C. Supernatants were used for protein concentration measurements. Pellets contained the intracellular melanin pigments, which were solubilized by incubation in 1 M NaOH for 2 h at 60°C. Extracellular melanin pigments were also solubilized, and absorbance was measured at 405 nm using a plate reader (SpectraFluor, Tecan). Standard curves were prepared for each experiment using synthetic melanin (0-250 μg/mL, Sigma). Data were normalized with respect to the protein content and then to the non-irradiated control.

### 4.9. Microscopic Observation and Quantification

Microscopic observations were performed using a fluorescence microscope (BX-61, Olympus, Tokyo, Japan) with the following filter sets: blue (450−490 nm, exciting filter BP 490), green (545 nm, exciting filter BP 545). Images were obtained with an Olympus CCD DP70 digital camera. Fluorescence intensity analysis (DHFDA and JC-1 expression) was performed using ImageJ version 1.52a (NIH, Bethesda, MD, USA). Data were normalized with respect to non-irradiated control values.

### 4.10. Statistical Analysis

Data are represented as the mean ± standard error of the mean (SEM) of at least three independent experiments. For statistical analysis, Analysis of variance (ANOVA) and Bonferroni *post hoc* tests were run using GraphPad Prism 5.00 (GraphPad Software, Inc., San Diego, CA, USA). Differences were considered to be significant when *p* ≤ 0.05. 

## Supplementary Materials

Supplementary materials can be found at https://www.mdpi.com/1422-0067/21/3/988/s1. Supplementary Figure S1. Visible light emission wavelengths and classification, and spectrum of blue light source.

## Abbreviations

CPD	Cyclobutane pyrimidine dimers
DHFDA	Dihydrofluorescein diacetate
DMEM	Dulbecco’s modified eagle medium
EDA	Extract from *Deschampsia antarctica*, EDAFENCE^®^
FBS	Fetal bovine serum
HDF	Human dermal fibroblasts
Irr	Irradiation
JC-1	5,5,6,6’-tetrachloro-1,1’,3,3’-tetraethylbenzimi-dazoylcarbocyanine iodide
LED	Light-emitted diode
MAPK	Mitogen-activated protein kinase
MITF	Microphthalmia-associated transcription factor
MTT	3-[4,5-dimethylthiazol-2-yl]-2,5-diphenyltetrazoliumbromide
ROS	Reactive oxygen species
SEM	Standard error of the mean
Trxs	Thioredoxins
UV	Ultraviolet
VIS	Visible light
