# Supplementary material for: Protective Effect of the Aqueous Extract of Deschampsia antarctica (EDAFENCE®) on Skin Cells against Blue Light Emitted from Digital Devices
Source: Int J Mol Sci. 2020 Feb 2;21(3):988. doi: 10.3390/ijms21030988 (PMC7038134; doi:10.3390/ijms21030988)
Supplement: Supplementary file 1 [file ijms-21-00988-s001.pdf]

(A)

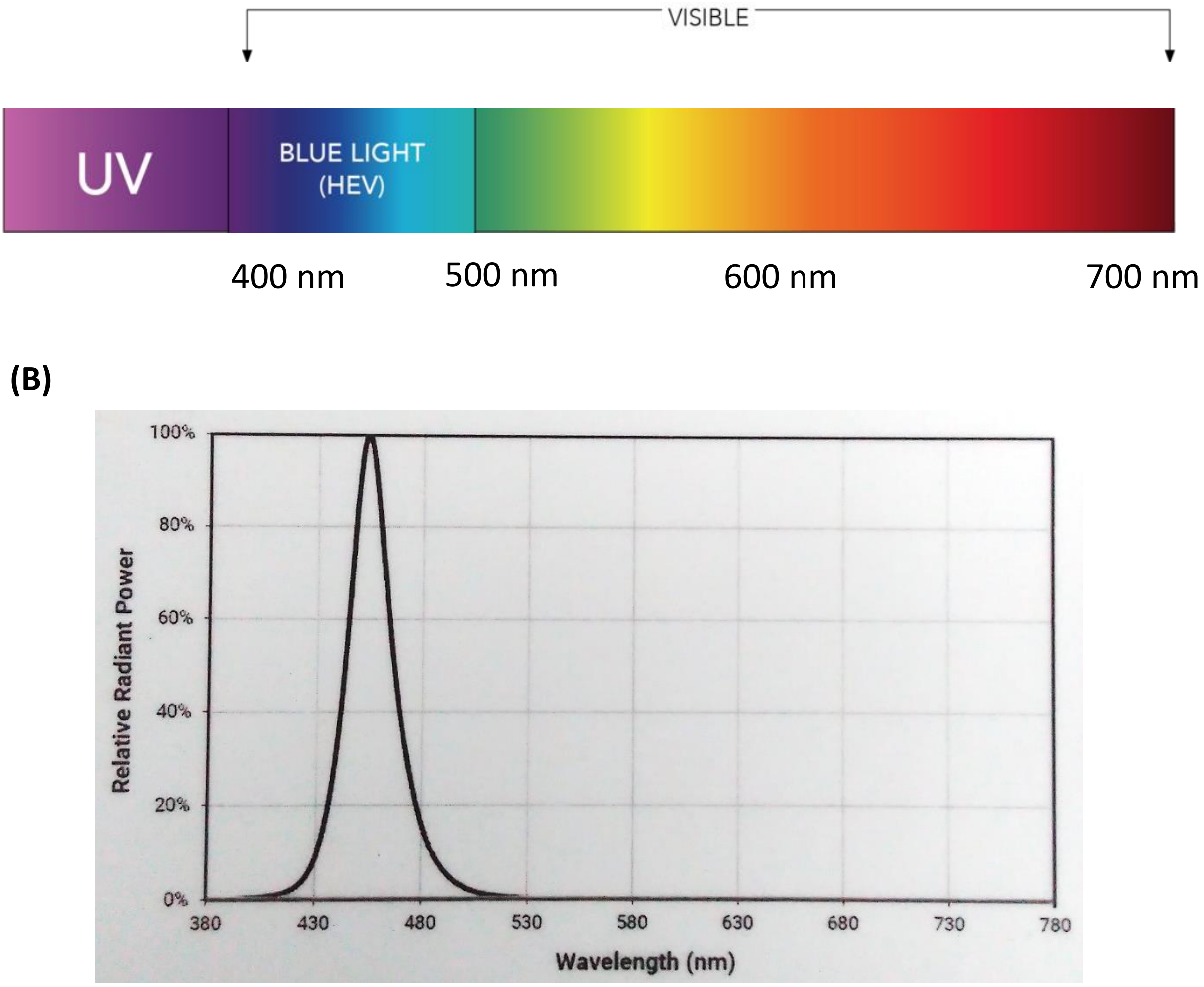

Supplementary Figure 1: (A) Visible light emission wavelengths and classification. Image adapted from Blue Light Exposed, 2018. **(B)** Spectrum of blue light source.
